# Cognitive Flexibility and Reaction Time Improvements After Cognitive Training Designed for Men Perpetrators of Intimate Partner Violence: Results of a Pilot Randomized Controlled Trial

**DOI:** 10.1007/s10896-021-00304-2

**Published:** 2021-08-05

**Authors:** Á. Romero-Martínez, F. Santirso, M. Lila, J. Comes-Fayos, L. Moya-Albiol

**Affiliations:** 1 Department of Psychobiology, University of Valencia, Avenida Blasco Ibañez, 21, 46010 Valencia, Spain; 2 Department of Social Psychology, University of Valencia, Valencia, Spain

**Keywords:** Cognitive training, Intimate partner violence, Neuropsychology, Risk of recidivism, Standard intervention programs

## Abstract

*Purpose* Current interventions for intimate partner violence (IPV) perpetrators are designed to reduce IPV recidivism by treating risk factors and increasing protective factors. However, these interventions pay less attention to cognitive functioning in IPV perpetrators and how these variables interfere with the future risk of recidivism. Therefore, the main objective of this research was to compare the effectiveness of Standard Intervention Programs for men who perpetrate IPV [SIP] + cognitive training vs SIP + placebo training in promoting cognitive improvements and reducing recidivism. Furthermore, we also aimed to assess whether changes in the risk of recidivism would be related to cognitive changes after the intervention. *Method* IPV perpetrators who agreed to participate were randomly allocated to receive SIP + cognitive training or SIP + placebo training. Several cognitive variables were assessed before and after the interventions with a complete battery of neuropsychological tests assessing processing speed, memory, attention, executive functions, and emotion decoding abilities. Moreover, we also assessed the risk of recidivism. *Results* Our data pointed out that only the IPV perpetrators who received the SIP + cognitive training improved their processing speed and cognitive flexibility after this intervention. Furthermore, these participants presented the lowest risk of recidivism after the intervention. Nonetheless, cognitive improvements and reductions in the risk of recidivism after the intervention were unrelated. *Conclusions* Our study reinforces the importance of implementing cognitive training to reduce risk of recidivism after SIP. Hence, these results might encourage professionals to incorporate neuropsychological variables in IPV intervention programs.

## Introduction

Approximately 30% of women worldwide have experienced some type of physical or sexual violence from their male partners at some point in their lives, whereas the percentage of males who have received physical violence from their female partners is around 15% (Cooper & Smith, 2012; Kellermann & Mercy, 1992; Rollè et al., 2018; Swan et al., 2008; World Health Organization, 2020). Recently, the percentage of maltreated women has increased considerably, as well as the number of men convicted for perpetrating intimate partner violence (IPV), due to the socioeconomic consequences of the SARS-CoV-2 pandemic (World Health Organization, 2020). Therefore, it is necessary to design effective intervention programs to prevent health consequences for victims and reduce the future risk of recidivism in IPV perpetrators.

Current interventions for IPV perpetrators are designed to reduce IPV recidivism by treating risk factors and increasing protective factors (Eckhardt et al., 2013; Lila et al., 2019; Murphy et al., 2017; Santirso et al., 2020). Most of these interventions mainly focus on reducing alcohol and/or drug misuse, attitudes toward violence and/or women, impulsivity, and anger (state and trait), among others. Moreover, these interventions try to promote, for example, self-esteem, social support, or awareness of IPV (Arce et al., 2020; Catalá-Miñana et al., 2013; Babcock et al., 2004; Lila et al., 2013, 2020; Martín-Fernández et al., 2018). However, current interventions and previous research in this field have paid less attention to cognitive functioning in IPV perpetrators (assessed by neuropsychological tests) and how these variables influence the future risk of recidivism. There is considerable interest in implementing cognitive training programs to treat cognitive deficits in several types of aggressive individuals. In fact, studies have pointed out that when psychotherapeutic interventions are reinforced with cognitive training, the future risk of reoffending is considerably reduced, in comparison with the application of the psychotherapy alone (Fisher et al., 2016; Genevsky et al., 2010; Twamley et al., 2003). Cognitive training might help schizophrenic patients to process information as quickly and accurately as possible. Therefore, after intensive cognitive training programs (daily for six or more months), these patients could present an enhancement of their cognitive functioning that would allow them to benefit more from the knowledge offered in psychotherapy. Although only a small percentage of IPV perpetrators suffer from schizophrenia (Yu et al., 2019), it could be particularly important to develop cognitive training programs for aggressive individuals with different cognitive deficits and/or alterations, thus reinforcing patients’ adherence to psychotherapeutic treatment and reduce the future risk of recidivism.

As pointed out for the somatic marker hypothesis (Damasio et al., 1991), cognitive deficits combined with a poor emotion processing system would lead individuals to choose reinforcements without anticipating the negative or positive consequences of their decisions and/or actions. In fact, this might be the result of failing to process available environmental information to foresee the consequences of their behavior (Sun et al., 2015). This is congruent with the proposal of social processing information, which establishes that IPV might be explained, at least in part, by the existence of hostile attributions, marital distortions, and cognitive deficits, among others, in IPV perpetrators (Agnew & South, 2014; Gracia et al., 2020; Murphy et al., 2014; Taft et al., 2017). These cognitive alterations or misattributions in interpreting marital situations or conflicts might facilitate IPV perpetration under certain ambiguous circumstances. Hence, based on previous evidence indicating that many IPV perpetrators present cognitive alterations, it is particularly important to specifically determine the degree of IPV perpetrators’ cognitive and/or social information processing deficits, in order to reinforce standardized intervention programs for IPV perpetrators with cognitive training interventions.

Regarding the cognitive functioning of IPV perpetrators, the deficits that have attracted the most interest, due to their importance in the regulation of behavior, are those related to executive functioning. In this regard, studies have shown that, in general, IPV perpetrators would present alterations at the level of cognitive flexibility (ability to adapt to a changing environment), decision-making, inhibition capacity, processing speed, and verbal abilities (e.g., phonological, and semantic fluency, abstraction, comprehension, etc.) rapidly and successfully. In addition, they have also shown alterations in attention, as well as a low capacity for abstraction and mnemic abilities (e.g., working memory and long-term memory) (Horne et al., 2020; Romero-Martínez & Moya-Albiol, 2013; Romero-Martínez et al., 2016a, 2016b, 2019a, 2019b). These alterations are especially pronounced if IPV perpetrators present higher consumption of alcohol and/or other drugs (Lila et al., 2016; Vitoria-Estruch et al., 2017, 2018). Nonetheless, it is less clear whether IPV cognitive performance could be categorized as a deficit (performance below 2–2.5 standard deviations from the normative group) or simply represent slightly below-average performance. However, not all the studies found that diminished executive dysfunctions explain IPV proneness. One study concluded that better planning abilities moderated the association between alcohol consumption and IPV perpetration during the past year (Schumacher et al., 2013). In any case, all of this information would be important in designing new interventions.

Analysis of IPV perpetrators’ emotional functioning suggests the existence of alterations in the ability to decode emotions based on facial expressions (Romero-Martínez & Moya-Albiol, 2013). This is a source of basic information that humans need in order to understand another individual’s perspective (perspective taking) and feel what someone else feels (affective arousal). Thus, a misunderstanding about facial emotion expressions, for example, when dealing with ambiguous emotional stimuli, could lower the threshold for reacting violently because these stimuli might be interpreted as hostile. Therefore, decoding emotions would be indirectly related to moral reasoning, prosocial behavior, and mood, among others (Balconi & Canavesio, 2016; Schipper & Petermann, 2013). Thus, alterations in the ability to decode facial emotion expressions could interfere with behavior control by interpretating ambiguous emotional facial expressions as threatening, with individuals presenting a coherent pattern of behavior response to this hostile interpretation, such as violence.

As far as we know, only two studies have assessed whether IPV perpetrators showed changes in cognitive and/or emotional functioning after implementing standard intervention programs. In fact, the first one was an uncontrolled study without cognitive training (Romero-Martínez et al., 2016b). The authors concluded that participants who completed the program showed improvements in their cognitive flexibility and emotion-decoding abilities, but they were unable to identify which intervention modules were associated with these improvements. The second study complemented the standard IPV perpetrator intervention with an individualized motivational plan in a randomized controlled trial (RCT). This research reported specific improvements in emotion-decoding abilities in participants who received the standard program complemented by the motivational plan (Romero-Martínez et al., 2019a). However, this intervention is not considered specific cognitive training. Unfortunately, there is a gap in the scientific literature assessing whether standard interventions, which are mainly cognitive- behavioral interventions, that are complemented with cognitive training would be more effective in reducing the risk of recidivism in IPV perpetrators, compared to standard intervention programs alone. Thus, it is necessary to conduct an RCT to find out whether cognitive training used in standard interventions promotes greater cognitive and emotion-decoding improvements in IPV perpetrators, as well as considerable reductions in the future risk of IPV recidivism. This research will allow us to clearly identify which exercises would be suitable to promote specific cognitive improvements.

The main objective of the present study was to assess the differences in the effects of standard intervention + cognitive training and standard intervention + placebo training on IPV perpetrators’ cognitive outcomes and risk of recidivism after ending both interventions. Studies have shown that intervention programs for IPV perpetrators promote cognitive and emotional improvements and a subsequent reduced risk of recidivism in IPV perpetrators who complete the interventions (Romero-Martínez et al., 2016a, 2016b, 2019a, 2019b), and that standard interventions with cognitive training demonstrate higher efficacy than standard interventions alone in producing improvements in violent individuals, such as schizophrenic patients (Fisher et al., 2016; Genevsky et al., 2010; Twamley et al., 2003). Therefore, our initial hypothesis was that standard intervention programs for men who perpetrate male-to-female IPV (SIP) + cognitive training would promote greater cognitive and emotion-decoding improvements and a lower risk of recidivism than SIP + placebo training. Moreover, we also aimed to assess whether changes in the risk of recidivism would be related to changes in neuropsychological performance. Based on previous results (Romero-Martínez et al., 2019a, 2019b), reductions in the risk of recidivism would be positively related to cognitive and emotion-decoding improvements.

## Method

### Participants

Participants were recruited from a mandatory treatment program for men convicted of IPV. The men received a suspended sentence with the condition that they had to attend this intervention program. This alternative was offered to men who were sentenced to less than two years in prison and had no previous criminal record (Lila et al., 2014, 2018). To be included in this study, participants had to be older than 18 years of age and have no physical (e.g., chronic pain, epilepsy…) or mental problems (e.g., schizophrenia, personality disorders…), no severe substance abuse problems, and no severe cognitive impairment due to brain damage and/or traumatic brain injury.

From an initial sample of 63 IPV perpetrators, only 51 IPV perpetrators completed the intervention. In fact, 12 participants left the intervention before it ended. Furthermore, 12 additional participants refused to participate during the second neuropsychological assessment. This refusal was due to the SARS-CoV-2 pandemic, which meant that a large number of participants could not physically visit our laboratories due to mobility restrictions in our country. Therefore, the final sample was composed of 39 participants included in the statistical analyses. Eleven of these participants were removed from the statistical analysis because six had missing information (e.g., neuropsychological tests) and five were outliers (> 2.5 SD from performance of their group). Therefore, the final sample was made up of 28 participants. All the IPV perpetrators who voluntarily agreed to participate in our study gave their informed consent to be included. Additionally, they were informed that the judicial system would not have access to the information collected during the interviews and study. The study was conducted in accordance with the Declaration of Helsinki, and the protocol was approved by the Ethics Committee (assigned codes: H1348835571691 and H1537520365110).

### Study Design

**Fig. 1:**
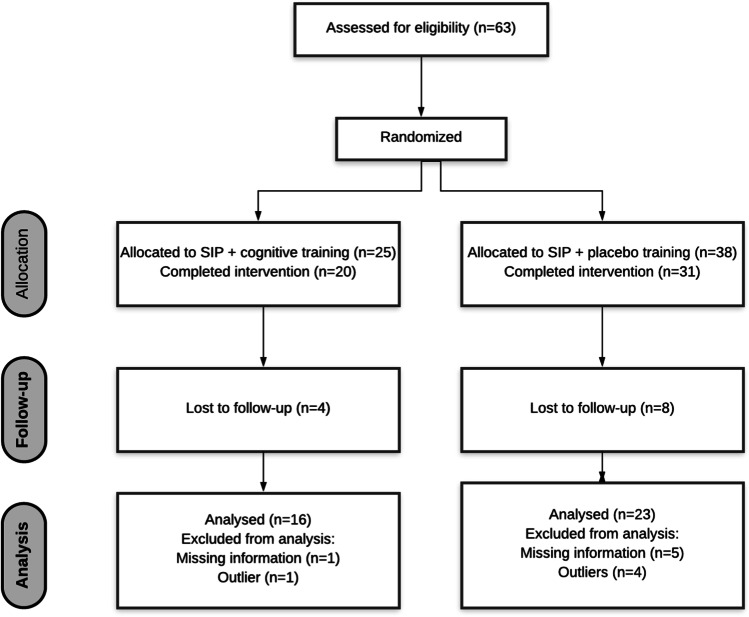
Consort diagram of the study

According to the functioning of the CONTEXTO program, and due to the SARS-COV-2 pandemic situation, it was only possible to select an initial number of 63 participants distributed in six therapy groups whose composition ranged from 9 to 11 men each. Before starting the initial assessment for randomization purposes, a coin-tossing method was employed to distribute the participants. This simple and unrestricted randomization procedure, along with the odd number of participants, explains the allocation of 25 and 38 participants to the groups (Fig. 1). As Schulz and Grimes (2002) indicated in their manuscript published in the Lancet, simple randomization usually produces discrepancies in the number of participants assigned to each group, although this should not be viewed as a potential study limitation because randomization entails unpredictability (Schulz & Grimes, 2002). Table 1 provides the description of each treatment condition, specifically information about the cognitive and placebo training.

**Table 1:** Characteristics of the cognitive and placebo training

	Standard batterer intervention program (SBIP) + cognitive training	Standard batterer intervention program (SBIP) + placebo training
Duration	35 group sessions (two days per week with each session lasting 2 h) + 31 sessions of cognitive training (two days per week with each session lasting 15 min)	35 group sessions (two days per week with each session lasting 2 h) + 31 sessions of placebo training (two days per week with each session lasting 15 min)
Cognitive training materials	Pen and paper and videos	–
Exercises in cognitive/placebo training	1. Selective attention 2. Verbal fluency (phonemic) 3. Alternating attention 4. Visual Memory (working memory and long-term) 5. Working memory (digits and letters) 6. Analysing racism and sexism in hidden figures (2016) 7. Language (comprehension) 8. Attention (sustained) 9. Analysing emotions of videos about evictions and homeless 10. Cognitive flexibility 11. Verbal fluency (semantic) 12. Analysing emotions in a TV-debate 13. Decision-making 14. Reasoning and problem-solving strategies 15. Analysing microfacial expressions in a TV-interview 16. Cognitive flexibility 17. Analysing emotions in main characters of The Shawshank Redemption (1994) 18. Working memory (digits) 19. Decision making 20. Analysing microfacial expressions in a TV-interview 21. Selective attention 22. Analysing emotions in main characters of Vikings (2013) 23. Verbal fluency (phonemic) 24. Verbal memory (working memory and long-term) 25. Analysing emotions in main characters The Truman Show (1998) 26. Selective attention 27. Visual memory (working memory and long-term) 28. Planification and problem-solving strategies 29. Analysing emotions in main characters Her (2013) 30. Selective attention 31. Verbal memory (working memory and long-term)	31 sessions alternating debate on current topics (40%), listening to relaxing music (30%), or teaching relaxation techniques (30%)

## Procedure

After providing their signed informed consent, each participant was assessed in three sessions (two initial sessions and a final session) in the psychobiology laboratories (Valencia, Spain). During the first session, we assessed participants’ suitability for inclusion in our study by excluding participants with physical or mental illnesses, and we also collected demographic variables, anthropometric variables, and drug misuse information. Participants included in the study were given an appointment the following day, specifically between 10 a.m. and 2 p.m., to avoid the effects of fatigue on the neuropsychological assessment. Initially, the assessment was conducted in a sound-proof room where we administered the neuropsychological battery and assessed the risk of recidivism. After the intervention programs (approximately 9 months later), the neuropsychological assessment was repeated (one week after the end of the intervention phase).

### SIP, Cognitive, and Placebo Training

To conduct this study, eight therapists, different from those who conducted the psychotherapeutic intervention, received a training course to acquire experience in the characteristics of the treatment condition to which each was assigned (e.g., how to administer worksheets, how to solve problems with participants, how to direct debates, showing debate strategies, learning relaxing strategies…). These therapists had at least one year of experience with IPV interventions. They were equally distributed in each condition and were blind to the condition, assuming that their colleagues carried out the same intervention program. Furthermore, we avoided contact between therapists, and they only received feedback from the main researchers in this project.

As described above, the psychotherapeutic intervention was conducted by other therapists different from those who performed the cognitive training or placebo. This intervention was initially designed to introduce basic concepts about IPV and address how IPV perpetrators make attributions about their responsibility, provide training in changing cognitive hostile schemas and techniques to manage emotions, teach positive communication and negotiation skills in intimate relationships, empathy, and awareness of IPV consequences for victims, discuss gender roles, among others, and, finally, prevent recidivism by consolidating the learning objectives (for further details, see Lila et al., 2018).

Based on previous scientific literature (Darmedru et al., 2017; Howren et al., 2014; Pedrero-Pérez et al., 2011; Seccomandi et al., 2020), particularly to avoid fatigue and boredom, the tasks used in each session alternated in their degree of difficulty and the time needed to carry them out. In other words, easier tasks were alternated with more complex tasks and tasks with different time requirements. The duration of each cognitive training session and the length of the rehabilitation program were based on the characteristics of the CONTEXTO intervention program (see for further details Lila et al., 2018). That is, it was necessary to carry out cognitive training on the same days that they received the CONTEXTO sessions because a large number of participants had limited time to attend during the rest of the week. Thus, we concluded that it would be appropriate to establish 15 min of cognitive training after 60 min of SIP intervention, followed by another 60 min of SIP. This would allow participants to benefit from an intervention program without interfering with the main functioning of the CONTEXTO program. Furthermore, we previously demonstrated that this psychotherapeutic program was associated with certain cognitive improvements (Romero-Martínez et al., 2016a, 2016b, 2019a, 2019b). Moreover, previous studies have included interventions lasting 15 min per day that were successfully associated with cognitive improvements, particularly in executive functioning (Nouchi et al., 2012; Shiran & Breznitz, 2011). In any case, our intervention lasted 30 min per week, which we decided would be the best length for practical reasons, thus increasing the possible benefits of the cognitive training.

The instruments used for the cognitive training were pencil and paper tests previously validated and standardized in the Spanish population (Marrón et al., 2011). Specifically, the authors provided extant material for performing cognitive rehabilitation, as well as links to advice about carrying out cognitive rehabilitation programs. In fact, they provided examples of materials for treating several cognitive domains, such as speed processing, attention, memory, language, and/or executive functioning. Specifically, for working on basic processes such as attention, we provided worksheets on completing visual stimuli, indicating congruent or incongruent stimuli based on a basic pattern, recognizing and/or identifying patterns, planning the schedule of a family based on certain characteristics, providing a social dilemma in order to stimulate participants to offer alternatives to certain problems, etc. For the rehabilitation of emotion-decoding abilities, we employed the material recommended in the scientific literature (Teding van Berkhout & Malouff, 2016). In this regard, the materials used to promote empathic capacities could be grouped as follows: segments of films with emotional content, where participants have to interpret the intentions or feelings of the main characters, detect facial expressions of emotions, etc.

For the placebo intervention, we concluded that the best alternative to cognitive training was to alternate several activities somewhat, but not completely, from materials and/or activities in the cognitive training. Specifically, three types of activities were employed: debating about different topics not directly related to IPV, training in relaxing exercises, or listening to music, alternating activities to simulate the dynamic of cognitive training.

In each session of the rehabilitation program, a specific cognitive or empathy-related domain (e.g., memory, executive functions, etc.) is worked on (see Table 1).

Finally, it should be kept in mind that therapists registered participants’ adherence by recording their attendance and/or attitude towards therapy. This was particularly important because if participants failed to attend a session, it would be necessary to recover it during the following sessions. Therefore, all the participants received the same number of cognitive training sessions. Moreover, therapists offered constant feedback to participants to achieve previously established session goals (e.g., number of complete worksheets assigned per session). This would allow all the groups to complete the same goals. Additionally, fidelity in running the RCT was ensured through biweekly supervision by the main researcher in the study (supervisor) after reviewing the sessions in person through a one-way mirror. In fact, the main role of the supervisor, who was not blind to the study groups, was to ensure that the therapists did their job as they were trained and resolve any doubts they might have.

## Measures

### Verbal and Non-verbal Abilities

We employed the Kaufman Brief Intelligence Test (K-BIT) (Kaufman & Kaufman, 1997) to assess verbal and non-verbal abilities. This test consists of two subtests: vocabulary and matrices. The vocabulary subtest includes two parts, one dedicated to expressive vocabulary (45 items) and the other dedicated to definitions (37 items). The matrices consist of series of drawings and abstract Figs. (48 elements).

### Working Memory

Digits (direct and inverse) is a subscale of the Wechsler Intelligence Scale-III (WAIS-III) that assesses short-term memory, attention, and concentration. On this test, participants are asked to repeat the digits in direct and reverse order (Wechsler, 1999). For this test, the total score was considered (direct order + indirect order).

### Processing Speed and Attention

We employed Conners’ Continuous Performance Test-III (CPT-III) to assess sustained visual and auditory attention, sequencing, and processing speed. It is a computerized test with an administration time of around 14 min. Participants have to press the space bar on the computer when any letter except "X" appears on the screen. The scales on this test are inattention, impulsivity, sustained attention, and vigilance. For this study, we considered reaction time (milliseconds, ms), number of omissions, commissions, and perseverations (Conners, 2015).

### Executive Functioning

For F-A-S verbal phonemic fluency, participants have to verbalize out loud as many words as possible that begin with F, S, and A for 60 s for each letter. A total score is obtained by adding one point for each correct response. Moreover, to assess verbal semantic fluency, participants are asked to provide as many animal names as they can for 60 s. One point is assigned for each correct animal name evoked in that time interval. In both cases, higher scores indicate better verbal fluency (Del Ser Quijano et al., 2004).

Wisconsin Card Sorting Test (Heaton, 1993). This test consists of two games with 64 cards each. The cards are made up of the combination of three kinds of attributes: shape (triangle, star, cross and circle), color (red, blue, green, and yellow), and number (one, two, three, or four elements). Participants have to learn the rules of the game, so that they can match the present card with one of the four possibilities that appear at the top of the screen.

### Emotion Decoding

The *Eyes Test* measures emotion-decoding abilities by identifying the emotion that best represents a series of photographs of the eye region, exactly 36, of different men and women. From a set of four options, participants have to choose the one that best describes the feelings or thoughts in each photograph. For this study, we employed the total score by adding up the correct responses. The maximum score is 36, and the minimum is 0, with higher scores indicating better emotion-decoding abilities (Baron-Cohen et al., 2001).

### Risk of Recidivism

For the assessment of the risk of recidivism in violence against women in intimate relationships, we use the Spouse Assault Risk Assessment (SARA) (Kropp et al., 1995), suitably adapted to Spanish (Andrés-Pueyo et al., 2008). This instrument consists of 20 items, classified on a 3-point scale (0 = absent; 1 = possibly present; and 2 = present). In fact, this instrument was implemented by therapists with expertise in this field who were different from those who conducted psychotherapy and cognitive training. This group of experts completed this tool mainly based on their observations of IPV perpetrators’ functioning during interventions and after the intervention concluded. Items include the main risk factors for this type of violence (e.g., use of weapons and/or death threats, attitudes that support or condone intimate partner violence, violations, and violations of restraining orders). A total score was obtained by summing the scores on the items, ranging from 0 (minimum risk or absence) to 40 (imminent risk of recidivism). Exclusively in this study, we only employed items that measured dynamic risk factors (e.g., recent use of weapons or death threats, drug misuse, work problems…), avoiding items that assess static factors (e.g., previous history of violence against family members, previous history of violence against strangers…).

### Drug Use

Alcohol use was measured with the alcohol use disorders identification test (AUDIT), adapted to Spanish (Contell-Guillamón et al., 1999; Saunders et al., 1993). This test was composed of 10 self-report items, rated on a scale ranging from 0 (never) to 4 (daily or almost daily). A total score was obtained by summing the scores on all the items, with a higher score indicating higher alcohol consumption.

Regarding cannabis and cocaine use, we employed the Spanish adaptation of the Severity Dependence Scale, asking about each drug (Miele et al., 2000; Vélez-Moreno et al., 2013). This test contains five items rated on a scale from 0 to 3. We obtained a total score by summing the scores on all the items, with higher scores indicating worse cannabis or cocaine consumption.

### Data Analysis

Before performing parametric or non-parametric analysis, the Shapiro–Wilk test was performed to assess whether the data had a normal distribution. After checking and verifying the assumption of normality (*p* < 0.05), we carried out parametric tests for statistical analysis. Thus, we performed *t*-tests and chi-square tests to study differences between groups on demographic variables, drug misuse, and the neuropsychological baseline (before intervention) assessment. Furthermore, we employed t-tests for attrition analysis. Moreover, we calculated the effect size using Cohen’s d (Cohen, 1988).

The effectiveness of the intervention in the total sample for the neuropsychological variables and risk of recidivism was evaluated with general linear model repeated-measures ANOVA, with ‘time’ (before and after intervention) as within-subject factor. To examine group effects, repeated-measures ANOVAs were conducted, with ‘time’ as within-subject factor and ‘type of intervention’ (cognitive vs placebo intervention) as between-subject factor. For significant results, partial eta-squared (η_p_^2^) was reported as a measure of the effect size. Furthermore, we also provided observed power. Additionally, we calculated treatment outcome (risk of recidivism and neuropsychological treatment) as the difference between the post and baseline intervention scores. Then, we assessed group differences by conducting t-tests for each of the previously mentioned variables. Pearson correlation coefficients were calculated to assess relationships between change scores (risk of recidivism and neuropsychological variables). Due to the sample size and the number of comparisons, Bonferroni correction for multiple comparisons was applied. Therefore, only correlations with a p value lower than 0.005 were considered significant.

Statistical analyses were performed using IBM SPSS (Version 26.0; IBM SPSS), and *p* ≤ 0.05 was considered significant. Average values are expressed as mean ± standard deviation.

## Results

Groups did not differ on the demographic variables (see Table 2). Moreover, there were no differences between groups in their drug misuse (e.g., alcohol, cannabis, and cocaine).

**Table 2:** Mean ± SD of anthropometric and demographic variables of participants

Demographic variables		Pre-intervention			
		Training (n = 14)	Control (n = 14)	T-test/Chi-square	Significance
Age (years)		46.57 (5.92)	42.29 (7.24)	1.71	0.098
Educational level	Primary	36%	36%	2.90	0.407
	Secondary/upper level	64%	64%		
Marital status	Married	36%	50%	1.33	0.721
	Separated/divorced	63%	50%		
Nationality	Spanish	86%	71%	6.18	0.403
	Other	14%	29%		
Working status	Yes	57%	78%	1.47	0.225
	No	43%	22%		
*Drug misuse*					
Alcohol		4.28 (4.28)	6.71 (5.76)	1.26	0.217
Cannabis		0.42 (1.60)	0.78 (2.00)	0.52	0.607
Cocaine		1.42 (3.95)	1.14 (2.76)	0.22	0.827

With regard to attrition analysis, it is important to highlight that no differences were found between participants who were removed from the study and those who were finally included, specifically, on age (*t*_61_ (50.80) = 0.24, *p* = 0.81), marital status (X^2^ (3) = 1.05, *p* = 0.78), nationality (X^2^ (1) = 1.16, *p* = 0.28), educational level (X^2^ (3) = 0.56, *p* = 0.91), and/or employment status (X^2^ (1) = 0.88, *p* = 0.35). Furthermore, there were no significant differences between those who completed the intervention and those who dropped out or whose data were lost, on IQ (*t*_61_ =  − 0.48, *p* = 0.63), Digit span WAIS-III (*t*_61_ =  − 1.11, *p* = 0.27), CPT-III Omissions (misses) (*t*_61_ =  − 0.75, *p* = 0.45), CPT-III Commissions (false alarms) (*t*_61_ =  − 0.49, *p* = 0.62), CPT-III Perseverations (*t*_61_ = 0.19, p = 0.84), FAS phonemic (*t*_61_ = 0.23, *p* = 0.82), FAS semantic (*t*_61_ =  − 0.58, *p* = 0.56), WCST perseverative mistakes (*t*_61_ = 0.19, *p* = 0.84), WCST Number of categories completed (*t*_61_ =  − 1.22, *p* = 0.25), Eyes test (*t*_61_ = 0.40, *p* = 0.69), and SARA (*t*_61_ =  − 0.27, *p* = 0.79).

### Before Cognitive Training

Regarding cognitive functioning, groups did not differ on IQ (*t*_26_ = 0.86, *p* = 0.40, *d* = 0.32), Digit span WAIS-III (*t*_26_ =  − 1.09, *p* = 0.28, *d* = 0.43), CPT-III reaction times (ms) (*t*_26_ = 1.35, *p* = 0.18, *d* = 0.52), CPT-III omissions (*t*_26_ = 0.73, *p* = 0.47, *d* = 0.28), CPT-III commissions (*t*_26_ =  − 1.27, *p* = 0.21, *d* = 0.48), CPT-III perseverations (*t*_91_ = 1.02, *p* = 0.16, *d* = 0.30), FAS phonemic (*t*_26_ = 0.28, *p* = 0.78, *d* = 0.11), FAS semantic (*t*_26_ = 0.12, *p* = 0.90, *d* = 0.05), WCST Perseverative mistakes (*t*_26_ = 1.33, *p* = 0.19, *d* = 0.50), WCST Number of categories completed (*t*_26_ = 0.18, *p* = 0.84, *d* = 0.08), or the Eyes test (*t*_26_ =  − 0.09, *p* = 0.93, *d* = 0.03). Furthermore, there were no significant differences between groups on risk of recidivism (*t*_26_ =  − 1,23, *p* = 0.23, *d* = 0.08).

### Effectiveness of the Intervention Program in Eliciting Neuropsychological and Risk of Recidivism Changes

There was a significant effect of ‘time’ in the total sample on CPT-III reaction time (which measures visual and auditory attention), *F*(1, 27) = 7.82, *p* = 0.009 *η*_p_^2^ = 0.23, *d* = 0.77. After analyzing each group separately, within-group comparisons only revealed significant effects of ‘time’ on the CPT-III reaction time in IPV perpetrators who received the cognitive training, *F*(1, 13) = 20.33, *p* = 0.001, *η*_p_^2^ = 0.61, *d* = 0.99. This group of IPV perpetrators presented lower reaction times after the intervention program. Furthermore, there was a significant ‘time x group’ effect on CPT-III reaction times, *F*(1, 26) = 4.33, *p* = 0.048 *η*_p_^2^ = 0.14, *d* = 0.52, although there were no post-hoc significant differences between groups. Finally, there were significant differences between groups in change scores (post-treatment–pre-treatment measurements). In fact, IPV perpetrators who received the cognitive training presented a greater decrease (− 91.33 ± 75.79) than IPV perpetrators who received the placebo training (− 15.99 ± 112.34) (see Table 3).

**Table 3:** Changes after intervention (neuropsychological intervention and risk of recidivism)

Cognitive domain	Test	Pre-intervention		Post-intervention		F ANOVA	η_p_^2^
		Training (n = 14)	Control (n = 14)	Training (n = 14)	Control (n = 14)		
Verbal and non-verbal abilities	K-BIT	97.42 (12.91)	93.71 (9.58)	–			
Working memory	Digit span WAIS-III	12.76 (3.70)	14.31 (3.49)	14.36 (5.22)	14.31 (3.40)	0.41	0.03
Processing speed	CPT-III reaction times (ms)	475.25 (64.11)	439.74 (74.32)	383.92 (67.53)	423.74 (66.14)	4.33*	.14
Attention	CPT-III Omissions (misses)	2.83 (5.98)	1.59 (2.09)	2.46 (5.88)	3.99 (7.74)	0.02	0.00
	CPT-III Commissions (false alarms)	23.13 (16.60)	31.35 (17.59)	13.02 (15.26)	25.17 (16.10)	0.02	0.00
	CPT-III Perseverations	0.42 (1.03)	0.02 (0.07)	0.12 (0.21)	0.10 (0.26)	0.01	0.00
*Executive functions*							
Verbal fluency	FAS phonemic	28.92 (12.57)	27.54 (11.72)	31.45 (12.77)	30.50 (8.54)	0.01	0.00
	FAS semantic	16.86 (5.14)	16.57 (6.90)	18.00 (4.09)	14.86 (3.27)	0.01	0.00
Cognitive flexibility (WCST)	Perseverative mistakes*	36.14 (26.73)	25.64 (12.73)	12.85 (6.28)	18.42 (15.17)	5.18*	0.17
	Number of categories completed	3.57 (1.74)	3.42 (2.06)	5.36 (1.44)	4.21 (2.01)	2.37	0.08
Emotional decoding abilities	Eyes test	20. 07 (4.37)	20.23 (4.86)	18.64 (5.04)	18.29 (3.87)	0.34	0.01
Risk of recidivism		8.50 (3.29)	10.00 (3.13)	5.923 (1.77)	9.50 (3.06)	5.32*	0.17

Regarding executive functioning, there was a significant effect of ‘time’ in the total sample on the WCST perseverative mistakes, *F*(1, 27) = 17.47, *p* < 0.001, *η*_p_^2^ = 0.39, *d* = 0.98, and the WCST number of categories completed, *F*(1, 27) = 14.93, *p* = 0.001, *η*_p_^2^ = 0.36, *d* = 0.96. Within-group comparisons only revealed significant effects of ‘time’ on WCST perseverative mistakes and WCST number of categories completed in IPV perpetrators who received the SIP + cognitive training, *F*(1, 13) = 12.81, *p* = 0.003, *η*_p_^2^ = 0.49, *d* = 0.91 and *F*(1, 13) = 17.94, *p* = 0.001, *η*_p_^2^ = 0.58, *d* = 0.97. In fact, these participants made fewer mistakes and completed more categories after the intervention. Regarding the ‘time × group’ effect, it was only significant for WCST perseverative mistakes, *F*(1, 26) = 5.18, *p* = 0.031 *η*_p_^2^ = 0.16, *d* = 0.60, but post-hoc analysis did not reveal significant differences between groups (see Table 3). Moreover, the assessment of change scores revealed significant differences between groups in WCST perseverative mistakes (*t*_16.05_ =  − 2.27, *p* = 0.037, *d* = 0.86), with IPV perpetrators who received the cognitive training (− 23.78 ± 24.86) making fewer mistakes than those who received placebo treatment (− 7.78 ± 8.56).

There was a significant effect of ‘time’ on risk of recidivism in the total sample, *F*(1, 27) = 10.08, *p* = 0.004 *η*_p_^2^ = 0.27, *d* = 0.86. After analyzing each group separately, within-group comparisons only revealed significant effects of ‘time’ on SARA in IPV perpetrators who received the SIP + cognitive training, *F*(1, 13) = 15.54, *p* = 0.002, *η*_p_^2^ = 0.55, *d* = 0.95. This group of IPV perpetrators presented a lower risk of recidivism after the intervention program. Furthermore, there was a significant effect of ‘time × group’ on SARA, *F*(1, 26) = 5.32, *p* = 0.029 *η*_p_^2^ = 0.17, *d* = 0.60. Post-hoc analysis revealed that IPV perpetrators who received the cognitive training presented less risk of recidivism than those who received the placebo program (see Table 3). Finally, the assessment of the change scores revealed significant differences between groups (*t*_26_ =  − 2.30, *p* = 0.029, *d* = 0.85), with IPV perpetrators who received the cognitive training presenting greater reductions in the risk of recidivism (− 2.57 ± 2.44) than those who received the placebo intervention (− 0.50 ± 2.31).

### Relationships Between Risk of Recidivism and Neuropsychological Performance After Treatment

In the correlation analysis, no significant associations were found between risk of recidivism and the neuropsychological variables, with significance equal to or below 0.001.

## Discussion

The present study demonstrated that only the IPV perpetrators who received the SIP + cognitive training improved their processing speed on CPT-III, made fewer perseverative mistakes, and completed more categories after the intervention program. Furthermore, these participants presented the lowest risk of recidivism after the intervention. Nonetheless, cognitive improvements and a reduction in the risk of recidivism after the intervention were unrelated. We initially assessed whether the two groups of treatment conditions differ on the neuropsychological variables and risk of recidivism after the end of each treatment. In this regard, the findings obtained in this study partly support our initial hypothesis. That is, SIP reinforced with cognitive training was associated with greater cognitive improvements than SIP + placebo training. Nevertheless, improvements were only found in processing speed and cognitive flexibility, as well as reductions in the risk of recidivism, whereas participants did not show changes in working memory, attention, verbal fluency, or emotion decoding.

It seems logical to conclude that the content of the SIP combined with cognitive training may improve the IPV perpetrators’ ability to attend to relevant stimuli through an accurate and slow assessment, compared to IPV perpetrators without cognitive training. Moreover, this intervention also included problem-solving and decision-making training applied to solving problems in the domestic context (SIP), along with several pen and paper exercises to improve cognitive flexibility. Therefore, this might explain improvements in processing speed and cognitive flexibility (assessed with the WCST). Nevertheless, the absence of improvements in working memory, attention, verbal fluency, and/or emotion decoding might be explained by the low number of exercises dedicated to these cognitive domains. For example, in comparison with cognitive flexibility, SIP did not include exercises to promote these domains. In fact, previous studies that reported improvements in each of these domains applied intensive training for a minimum of 1 h per day for approximately 5 weeks, dedicated exclusively to a specific cognitive domain (working memory or attention) (Fisher et al., 2016; Genevsky et al., 2010; Twamley et al., 2003). Moreover, the lack of improvement in emotion decoding could be explained by the absence of changes in attention and/or working memory. Previous research in this field pointed out the significant and positive relationship between performance on attention tests and emotion-decoding abilities (Romero-Martínez & Moya-Albiol, 2013; Romero-Martínez et al., 2016a, 2019a, 2019b; Vitoria-Estruch et al., 2018). Furthermore, for decoding and recognition of facial expressions, participants do not need new ways of thinking (e.g., problem-solving abilities, planification, cognitive flexibility…), but they also focus their attention on faces for long periods of time, process several types of information (e.g., verbal, and non-verbal information), and pay close attention to microfacial expressions. Even so, this fact should be considered in future research on implementing cognitive training. Additionally, it would be necessary to reconsider the material employed to foment empathic and emotion-decoding abilities. We also consider it particularly important to acknowledge that there are a few differences between the active and placebo groups that might interfere with cognitive changes. Thus, it would be important to consider other potential control groups to compare with cognitive training.

Regarding the second aim of this paper, that is, to assess the relationship between changes in risk of recidivism and neuropsychological performance, we failed to find significant associations between these variables. The main problem was the reduced sample size, which forced to us to apply Bonferroni correction for multiple comparisons. Even though the cognitive training in our study led to reductions in risk of recidivism and improvements in specific cognitive domains, these changes were unrelated. Conversely, previous research demonstrated that cognitive improvements in IPV perpetrators involved a reduction in the risk of recidivism (Balconi & Canavesio, 2016; Romero-Martínez & Moya-Albiol, 2013; Romero-Martínez et al., 2016a, 2019a, 2019b; Schipper & Petermann, 2013; Vitoria-Estruch et al., 2017, 2018). Hence, this study should be replicated with a larger sample size and an extensive neuropsychological battery.

To the best of our knowledge, this is the first RCT to assess whether SIP combined with cognitive training for IPV perpetrators might promote improvements in several cognitive domains (assessed by neuropsychological tests). The main strength of this research is the study design (RCT) with a placebo control group (double blinded). Moreover, we employed a set of neuropsychological tests that present appropriate psychometric properties (e.g., test–retest reliability) and a carefully matched sample in terms of their demographic characteristics, IQ, or drug misuse. However, future studies should include a larger sample size and an extensive assessment of different cognitive domains. Additionally, we consider it necessary to include information about official recidivism in the follow-up after the intervention. Furthermore, characteristics of the cognitive training should be changed, for example, by increasing the length of each session (approximately 60 min) and holding daily sessions instead of two sessions per week. This would be made possible by providing training sessions through Apps on mobile phones or laptops. Finally, future studies should consider these limitations and implement a much larger controlled study than the current one.

Our study pointed out that the cognitive training specifically improved a few cognitive domains in IPV perpetrators. Moreover, our study supports the need for cognitive training in IPV interventions because the improvement in these variables might directly and/or indirectly reduce the future risk of recidivism. Thus, these results may lead clinicians and other professionals in psychology to design specific intervention programs based on IPV perpetrators’ needs. In fact, we provide a neuropsychological assessment with a detailed cognitive training program that can guide future research in this field. However, this study should be considered a pilot study due to its methodological limitations. Therefore, it is necessary to replicate these results in future studies before applying those conclusions in clinical practice.
